# Role of the Microbiota in the Modulation of Vaccine Immune Responses

**DOI:** 10.3389/fmicb.2019.01305

**Published:** 2019-07-03

**Authors:** Annalisa Ciabattini, Raffaela Olivieri, Elisa Lazzeri, Donata Medaglini

**Affiliations:** Laboratory of Molecular Microbiology and Biotechnology, Department of Medical Biotechnologies, University of Siena, Siena, Italy

**Keywords:** gut microbiota, microbiome, vaccines, immune system, next-generation sequencing

## Abstract

The human immune system and the microbiota co-evolve, and their balanced relationship is based on crosstalk between the two systems through the course of life. This tight association and the overall composition and richness of the microbiota play an important role in the modulation of host immunity and may impact the immune response to vaccination. The availability of innovative technologies, such as next-generation sequencing (NGS) and correlated bioinformatics tools, allows a deeper investigation of the crosstalk between the microbiota and human immune responses. This review discusses the current knowledge on the influence of the microbiota on the immune response to vaccination and novel tools to deeply analyze the impact of the microbiome on vaccine responses.

## Introduction

The term “human microbiota” refers to the complex communities of trillion symbiotic microbial cells including bacteria, archea, viruses and fungi harbored by each individual. The number of bacteria in the body is approximately of the same order as the number of human eukaryotic cells, as recently revised by the study of Sender’s group (Sender et al., 2016). The majority of them colonize the intestinal tract (gut microbiota), but microorganisms are present also in other areas including the skin, the airways, and the genitourinary tract. The term “human microbiome” generally refers to the collective genes harbored by these microbial cells. In the human gut, the microbiome includes about 3 million unique genes mostly from bacteria (Turnbaugh et al., 2007). These bacterial communities play a critical role in several body functions such as food digestion, synthesis of essential vitamins, and protection against pathogenic invaders (Kho and Lal, 2018).

The composition of the microbiota can be modulated by several factors throughout the course of life, and it is particularly unstable in the first 2 years (Tamburini et al., 2016). Factors such as gestational age, mode of delivery, diet, antibiotics exposure, probiotics and nutritional supplements use, hygiene conditions host genetics as well as the interaction with the immune system influence the composition of the microbiota. The first years of life is the phase when the early microbiota can shape the immune system and vice versa. During this period, commonly defined as “critical window,” the instability of the microbiota probably reflects the plasticity of the immune system in this time-limited period that permits the high variability of the microbial composition. Microbial imbalance, a state called “dysbiosis,” can predispose the body to disease (Shreiner et al., 2015; Kho and Lal, 2018). Many studies have highlighted the role of the gut microbiota in inflammatory bowel diseases (IBDs) (Machiels et al., 2014; Zuo and Ng, 2018), *Clostridium difficile* infection (Theriot et al., 2014), metabolic and neuropsychiatric disorders (Kho and Lal, 2018), rheumatoid arthritis, and other autoimmune diseases (Altmann et al., 2018) and of non-gut microbiota in diseases such as cystic fibrosis, chronic rhinosinusitis, periodontal, and chronic obstructive pulmonary disease and recently in central nervous system disorders (Wang and Kasper, 2014). The widely used concept of “dysbiosis” is, however, challenging in its definition because the known intra-variability and inter-variability of the human microbiota make difficult the distinctions between normal and abnormal microbial communities (Hooks and O’Malley, 2017). The knowledge of the dynamics underlying the interactions between the microbiota and the immune system is important for understanding the potential implications for human health.

In recent years, it has been shown that the microbiota can also influence the immune response to vaccination (Jamieson, 2015; Zimmermann and Curtis, 2018b) and that microbial alteration can impact the immune response to injectable and oral vaccines (Lynn et al., 2018). Human immune response and the microbial community develop in concert during the first months of life when the majority of vaccinations are given. For this reason, the composition of the early microbiota could potentially play an important role in the responses to vaccines. Evidence that the microbiota can influence vaccine responses comes from both pre-clinical and clinical studies (Table 1). With the use of germ-free (GF) or antibiotic-treated animals, impaired antibody responses to influenza vaccine have been observed, thus providing a demonstration of an effect of the microbiome on vaccination (Oh et al., 2014). In humans, Actinobacteria were shown to positively correlate with adaptive immune responses to systemic [Bacillus Calmette–Guérin (BCG), tetanus toxoid (TT), and hepatitis B virus (HBV)] and oral (polio) vaccination in Bangladeshi infants, whereas higher levels of Enterobacter were negatively associated (Huda et al., 2014). Other studies focused on the rotavirus vaccine (RVV) immune response confirmed a correlation between the infant gut microbiota composition and the vaccine responses (Harris et al., 2017; Harris V. et al., 2018).

**TABLE 1:** Correlations between microbiota and immune response to vaccination.

**Vaccine^a^**	**Host**	**Major findings**	**References**
Meningococcal serogroup B and C vaccines, the 13-valent pneumococcal conjugate vaccine; the hexavalent combination vaccine^b^, BCG	Infant mice	Antibiotic-driven dysbiosis leads to impaired antibody responses to vaccination	Lynn et al.,2018
TIV OPV	Mice	Correlation between early expression of TLR5 and the magnitude of the antibody response. Vaccine antibody responses in GF or antibiotic-treated mice were impaired but restored by oral reconstitution with a flagellated strain of *E. coli*	Oh et al.,2014
Live attenuated oral *Shigella dysenteriae* 1 vaccine	NHP (cynomolgus macaques)	The high level of diversity in the intestinal microbiota of macaques correlates with improved protection upon vaccination	Seekatz et al.,2013
Live cholera strain CVD 103-HgR	Children from low-income and high-income countries	Small bowel bacterial overgrowth (SBBO) could blunt the immune response to CVD 103-HgR	Levine,2010
Live attenuated oral Rotavirus vaccine	Healthy Ghanaian infants, RVV vaccinated	Intestinal microbiome composition correlates with RVV immunogenicity and may contribute to the diminished RVV immunogenicity observed in developing countries	Harris et al.,2017
	Indian infants	No significant differences in overall microbial community between responder and not-responder infants to RV vaccination. A mmodest inhibitory effect of co-administered OPV on the first dose of RV was observed	Parker et al.,2018
	Pakistan infants	RV response correlated with a higher relative abundance of bacteria belonging to *Clostridium* cluster XI and Proteobacteria, including bacteria related to *Serratia* and *E. coli*	Harris V. et al.,2018
Live attenuated oral *Salmonella typhi* Ty21a vaccine	Adult healthy volunteers	Differences in microbial composition or temporal stability observed among individuals displaying multiphasic cellular responses and not among individual able to mount a positive humoral response	Eloe-Fadrosh et al.,2013
OPV, BCG, TT, HBV	Bangladesh infants	High relative abundance of Actinobacteria, particularly of *Bifidobacterium*, can enhance responses to both oral and parenteral vaccines in infancy. Vaccine responsiveness can be improved by promoting intestinal Bifidobacteria and minimizing dysbiosis in infancy	Huda et al.,2014

*^*a*^Inactivated influenza vaccine (TIV); oral polio virus (OPV); Bacillus Calmette–Guérin (BCG); tetanus toxoid (TT); and hepatitis B virus (HBV). ^*b*^Against hepatitis B, diphtheria, tetanus, pertussis, Haemophilus influenzae type b, poliomyelitis virus.*

In this review, we discuss the interactions of the bacterial microbiota with the human immune system during the course of the life, with a particular focus on the potential role exerted on the immune response to vaccination.

## The Human Gut Microbiota

The gastrointestinal tract is a dynamic and semipermeable multilayer ecosystem, where different players crosstalk, forming a functional unit. Over 70% of the human microbiota lives in the gastrointestinal tract in a mutually beneficial relationship with its host. The gut microbiota is composed of 10^13^ to 10^14^ bacteria per gram of wet content, and it is characterized by a gradient in bacteria concentration steadily increasing from the gastric lumen to the small intestine up to the colon–rectum, where it reaches its maximum concentration (Ursell et al., 2012). Early gut colonizers include commensal facultative anaerobes such as Enterobacter and Enterococci followed by an increased relative abundance of strict anaerobes including *Bifidobacterium*, *Bacteroides*, and *Clostridium* (Matamoros et al., 2013; Hugon et al., 2015). The gut microbiota is dominated by the bacteria phyla Firmicutes and Bacteroidetes and to a lesser extent by Proteobacteria, Actinobacteria, and Verrucomicrobia (Bäckhed et al., 2012). Bacterial localization along the gastrointestinal tract is strongly influenced by nutrient distribution. Proteobacteria and Lactobacillales are present in the small intestine because monosaccharides, disaccharides, and amino acids present in this sector represent their main growth factors. Beyond the ileocecal valve, the microbial community changes, because most of the carbohydrates available are polysaccharides that Proteobacteria are not able to digest and use as a source of energy whereas *Bacteroides* and Clostridiales have enzymes that cut undigested polysaccharides. These fermentation processes produce acetate, propionate (Bacteroidetes), and butyrate (Firmicutes) that play an important role in maintaining a healthy gut. The microbial community also shows substantive differences between mucosal to lumen surfaces (Thursby and Juge, 2017): Enterobacteriaceae and *Bacteroides*, *Bifidobacterium*, *Streptococcus*, *Enterococcus*, *Clostridium*, *Lactobacillus*, and *Ruminococcus* genera are predominant in the luminal surface and can be identified in stool, whereas *Clostridium*, *Lactobacillus*, *Enterococcus*, and *Akkermansia* are the predominant genera detected in the mucus layer and epithelial crypts of the small intestine (Swidsinski et al., 2005).

Recent advances in culture-independent approaches and new techniques for viral enrichments from complex microbial samples have increased the knowledge on another important component of the microbiota, the “virobiota” and its genetic content (virome) (Virgin, 2014). Both eukaryotic viruses and bacteriophages are present in many host sites such as the intestine, oral cavity, urinary tract, and skin. The most abundant viruses associated with healthy human tissues are bacteriophages, including both lysogenic prophages and lytic phages that can establish a nonpathogenic relationship with the human host (Dalmasso et al., 2014). Populations of eukaryotic viruses in humans are heterogeneous, with distinct groups of viruses predominating in different tissues. Viruses in the gut mainly belong to the families Picornaviridae, Reoviridae, Caliciviridae, and Astroviridae, but members of the Adenoviridae, Picobirnaviridae, Herpesviridae, and Retroviridae families can also be found (Duerkop and Hooper, 2013). The lack of exhaustive information on virome underscores the need to dedicate more efforts to its characterization and to expand our knowledge of bacteriophages and their role in shaping host immunity.

The characterization of the interactions between the complex microbial community and the host is fundamental for understanding the role that the microbiota plays in health and disease, and potentially on the immune response to vaccination.

## The Crosstalk Between Microbiota and Immune System

The continuous and balanced interplay between the microbial flora and the host is essential to preserve the body’s normal functions. The immune system plays a critical role in maintaining homeostasis with resident microbial communities ensuring a mutualistic relationship. At the same time, the microbiota shapes the immune system during the early life and thereafter continues to modulate immunologic functions that are critical for the host physiology.

In the absence of microbial stimulation, the intestinal immune system is largely underdeveloped, both anatomically and functionally (McDermott and Huffnagle, 2014). For example, GF mice have a small number of intraepithelial lymphocytes and an inappropriate balance of helper T-cell subsets, with reduced production of immunoglobulin A (IgA) and antimicrobial peptides (AMPs). Other anatomic alterations are also present, such as immature mesenteric lymph nodes and less active Peyer’s patches (PPs) with small germinal zones, reduced mucus thickness with altered properties, elongation of the villi structures with atrophy of crypts, and defective angiogenesis. All these alterations are postulated to result from the lack of microbiota-derived signals as shown by the re-establishment of the normal organization of the intestinal immune system upon microbial stimulation (Sommer and Bäckhed, 2013). Moreover, murine neonatal T cells are skewed toward a Th2 phenotype, which exerts suppressive effects on Th1 cell-mediated immunity, thus allowing microbial homing and limiting potentially harmful pro-inflammatory responses (Adkins and Du, 1998). Also in humans, it has been shown that neonatal naïve CD4^+^ T cells have an intrinsic preference to differentiate into Th2 cells (Hebel et al., 2014). Various regulatory cells are also involved in the control of the immune homeostasis in order to tolerate the microbial colonization at mucosal sites at birth. In contrast, a state of dysbiosis promotes a strong Th1 displacement and an inflammatory state that can be involved in some diseases in adulthood (Tamburini et al., 2016).

**FIGURE 1:**
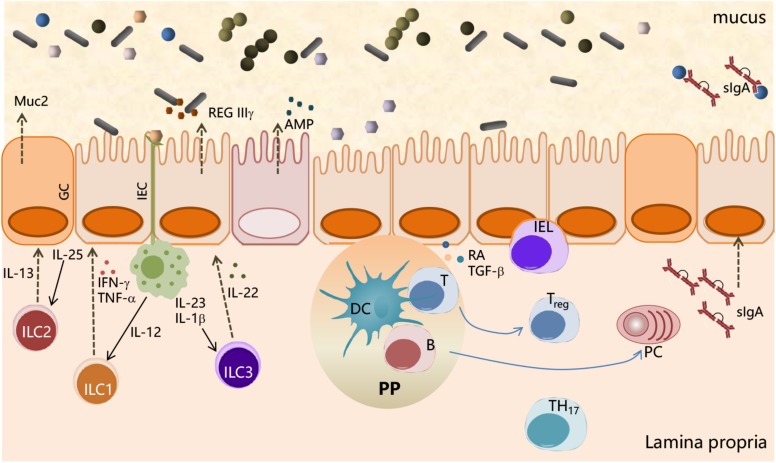
Interactions between the microbial community and the immune system at mucosal surfaces. Interactions between microbiota and host cells occur mainly at the intestinal epithelium surface, which constitutes the principal physical and chemical barriers for maintaining intestinal immune homeostasis. The gut microbiota is separated from the intestinal epithelium by a layer of mucus secreted by goblet cells (GCs). Microbe-associated molecular patterns (MAMPs), expressed on the bacterial surface, are recognized by pattern recognition receptors (PRRs), expressed by intestinal epithelial cells (IECs), and induce a variety of effects to block bacteria such as the production of the antimicrobial peptides (AMPs). IEC-released factors, such as retinoic acid (RA) and TGF-β, promote the development in the *lamina propria* of tolerogenic DCs that stimulate the differentiation of T cells into Treg. B cells differentiate into plasma cells (PCs) secreting IgA that translocate through the epithelium and are released into the mucus layer where control bacteria adhesion to host tissues. Macrophages, stimulated by signals such as flagellin, release IL-23, which in turn promotes the production of IL-22 by ILC3. IL-22 stimulates the release of RegIIIγ, an antimicrobial peptide produced by IECs. ILC2 contributes to the control of mucus by secreting IL-13, a cytokine that drives the differentiation of intestinal epithelial stem cells toward GC, which in turn produce mucin glycoproteins.

As schematically represented in Figure 1, the main interface between the host and microorganisms is represented by the intestinal epithelial cells (IECs); the microbiota communicates with the IECs through microbe-associated molecular patterns (MAMPs) and metabolic products. On the other hand, IECs are provided with specialized surface structures (microvilli, cilia, mucus production, and intercellular junctions) and a set of innate immune receptors, named pattern recognition receptors (PRRs), which recognize the MAMPs (Belkaid and Hand, 2014). PRRs, classified into toll-like receptors (TLRs), NOD-like receptors (NLRs), and retinoic acid-inducible gene I (RIG-I)-like receptors (RLRs) (Kamada et al., 2013), recognize microbial structures such as lipopolysaccharide (LPS) (TLR4), flagellin (TLR5 and NLRC4), lipoteichoic acid, bacterial lipoproteins, and peptidoglycan of Gram-positive bacteria (TLR2, Nod1, and Nod2).

The immune system mounts a series of innate and adaptive immune mechanisms aimed at reinforcing microbiota containment, barrier immunity, and tissue repair in a manner uncoupled from inflammation. Tolerance of the normal gut microbiota is a crucial element of intestinal homeostasis, requiring an extensive network of regulatory immune cells including T regulatory (Tregs) and tolerogenic dendritic cells (DC) (Belkaid and Harrison, 2017). Sensing of commensal microbiota through the TLR-MyD88 signaling pathway is a strategy applied by the immune system for maintaining host–microbial homeostasis (Rakoff-Nahoum and Medzhitov, 2007). For example, the surface polysaccharide A (PSA) produced by *Bacteroides fragilis* and recognized by the TLR2 mediates conversion of CD4^+^ T cells into Foxp3^+^ IL-10-producing Treg cells, promoting the immunologic tolerance in the gut (Round et al., 2011). Also, Clostridia species, especially *Clostridium* cluster IV (*C. leptum* group) and XIVa (*C. coccoides* group), are implicated in the development of local and systemic IL-10-expressing Foxp3^+^ Treg cells and the production of transforming growth factor-β1 in the large intestine (Atarashi et al., 2011). The expression of PRRs varies considerably in the different tracts of the gut as well as the composition of the resident microbiota. As a result, crosstalk between the microbiota and the immune system is different in the colon compared with the small intestine (Schuijt et al., 2013).

Furthermore, IECs release factors, such as retinoic acid and TGF-β, that promote the development in the *lamina propria* of antigen-presenting cells (APCs), both DCs and macrophages, with tolerogenic properties (Figure 1). APCs stimulated by signals, such as flagellin, release IL-23 that in turn promotes the expression of IL-22 by innate lymphocyte cells type 3 (ILC3). IL-22 stimulates the production of RegIIIγ, an AMP produced by IECs, which is retained in the mucus layer (Kinnebrew et al., 2012). ILC2 indirectly control microbiota colonization by secreting IL-13, a cytokine that drives the differentiation of intestinal epithelial stem cells toward goblet cells, which in turn produce mucin glycoproteins (Britanova and Diefenbach, 2017; Figure 1). These antimicrobial mechanisms are constitutively engaged by the immune system to prevent overgrowth of the colonizing microbes and monitoring the resident microbiota through a process called immune homeostasis (Abraham and Medzhitov, 2011).

IgA production is also influenced by the gut microbiota; indeed, the number of IgA-producing cells in the intestine is markedly decreased in GF mice (Fagarasan et al., 2010). The dominant inductive site for IgA B-cell responses in GALT are the PPs, in which the formation of GC plays a critical role for the development of affinity matured IgA B cells and the formation of long-lived plasma and memory B cells. Contrary to other inductive sites, the PPs consistently exhibit GC owing to the presence of the microbiota and of food antigens in the lumen. There are several factors that promote the IgA isotype switching, such as TGF-β, the proliferation-inducing ligand (APRIL), or B-cell activating factor (BAFF). In addition, nitrogen oxide produced by iNOS-expressing cells can promote IgA differentiation, possibly *via* regulation of TGF-β receptor II expression on B cells (Tezuka et al., 2011). Within the intestinal *lamina propria*, B cells secrete soluble IgA, which are subsequently transcytosed across epithelial cells, thereby controlling host–commensal interaction by binding to luminal microorganisms, thus preventing their translocation across the epithelial barrier (stratification) and preserving its integrity and functionality (Mathias et al., 2014). Beyond their well-known role in pathogen neutralization and clearance (Cerutti et al., 2011), SIgA selectively retro-transports bound antigens back into intestinal Peyer’s patches where immune complexes associate with DC resulting in the onset of immunomodulatory types of responses (Corthésy, 2013). IgA-deficient mice indeed show increased microbiota penetration of the intestinal barrier and elevated microbe-specific serum IgG (Suzuki et al., 2004; Figure 1).

Gut microbiota releases metabolic products, which can affect intestinal integrity and stimulate the production of cytokines with pro-inflammatory or anti-inflammatory activity (Hooper et al., 2012) and provides numerous nutritional benefits to the host, including vitamins and short chain-fatty acids (SCFAs). SCFAs are bacterial fermentation products that act by modulation of IECs and leukocyte development (Corrêa-Oliveira et al., 2016). Clostridial species are the major producer of SCFAs, and this is probably associated with their potent anti-inflammatory activity in experimental models. An anti-inflammatory role has been associated with members of the genus *Lactobacillus* that are poor producers of SCFAs but strong producers of lactic acid that, in turn, can be rapidly converted to butyrate (one of the most important SCFA) by other members of the microbiota (Wullt et al., 2007).

Also, bacteriophages play an important role in promoting the development and activity of the immune system through interactions with their host bacteria. They act as reservoir of genetic diversity, being vehicles for the genetic exchange among bacteria. Indeed, phages are carriers of virulence determinants, resistance genes, and metabolic pathways through the process of genetic transduction, with a consequent impact on host metabolism and immunity (Duerkop and Hooper, 2013; Chatterjee and Duerkop, 2018). Qualitative and quantitative alterations in the community of bacteriophages associated with the gut mucosa can influence the bacterial microbiota ecology and pathogen fitness; this could have profound effects on modulation of pro-inflammatory or anti-inflammatory responses (Zuo and Ng, 2018).

The crosstalk between the microbiota and the immune system takes place throughout the course of life and is influenced by different immune responsiveness at the extremes of age. Indeed, the immune system of elderly people is remodeled with fewer naïve cells, mainly owing to changes in progenitor cells and primary lymphoid organ involution, increase in dysfunctional memory cells, and altered innate immune response (Linton and Dorshkind, 2004; Pawelec, 2018), leading to greater susceptibility to infectious diseases and reduced responses to vaccination (Ciabattini et al., 2018a). At the same time, the immune system of elderly people is characterized by the progressive onset of a chronic, sterile, and low-grade inflammation, a set of phenomena described by the concept of inflammaging (Franceschi et al., 2000). In correlation with these phenomena of immunosenescence and inflammaging, a profound remodeling of gut microbiota occurs progressively with age (Biagi et al., 2013; Odamaki et al., 2016). Deterioration of physiological functions of the human body can contribute to the reduction of species such as Bifidobacteria in the gut microbiota, whereas inflammation may both induce an increase of facultative aerobes (i.e., Enterobacteriaceae, Enterococcaceae, and Staphylococcaceae, which can grow in an inflamed gut because they are relatively oxygen tolerant) and inactivate the strict anaerobic Firmicutes. Moreover, Enterobacteriaceae have the capability to secrete LPSs, which may act as an endotoxin, increasing inflammation in the gut (Rhee, 2014; Kumar et al., 2016).

In general, a reduced biodiversity and compromised stability of the intestinal microbiota with respect to younger individuals have often been reported in the elderly people, even if a large inter-individual variability in older subjects has been observed (Claesson et al., 2011). Moreover, the comparison of studies performed in subjects having different nationalities highlighted a certain country specificity in how the aging process impacts on the intestinal microbiota (Biagi et al., 2013), possibly related to differences in lifestyle and dietary habits.

In summary, the microbiota plays a key role in the development and modulation of the immune system throughout the life, and at the same time the immune system contributes to maintain microbial homeostasis; this interplay can be one of the factors that impact vaccine responses. Furthermore, the association between certain members of the microbiota and activation of specific arms of intestinal immunity, linked to lifestyle geographic and environmental factors, can play a critical role in the heterogeneity of disease prevalence and possibly in vaccine responses throughout the world.

## Potential Role of the Microbiota in the Immune Response to Vaccination

**FIGURE 2:**
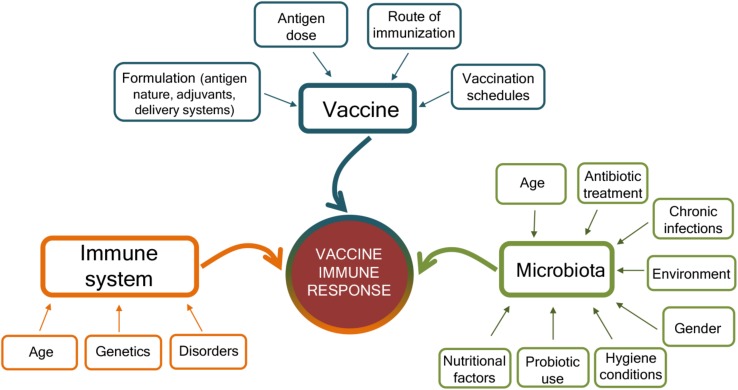
Factors affecting the immune response to vaccination. Immune responses to vaccination are affected by factors related to the vaccine, the host immune system, and the microbiota. The vaccine formulation (including the delivery system or adjuvant), the antigen dose, the route of vaccine administration (parenteral or mucosal), and the prime-boost strategies selected for the vaccination schedules deeply influence the host immune response to vaccination. At the same time, the host immune system is affected by age, genetic background, and possible disorders (allergy, autoimmunity, immunodeficiency, etc.). The host gut microbiota, susceptible to age, nutritional and environmental factors, gender, hygiene conditions, antibiotic treatment, use of probiotic, or chronic infections, also impact the capacity to respond to vaccination. Vaccination outcome is the result of the complex interplay of these different factors.

The mechanisms that can affect the response to vaccination are complex and include factors related to the vaccine, the host immune system, and the gut microbiota (Figure 2). The characteristics of the vaccine, including the vaccine formulation (i.e., vaccine delivery systems, adjuvants, and immunomodulators), the antigen nature (i.e., whole microorganism, purified protein, polysaccharide, and nucleic acid), the dose, the route of immunization (parenteral or mucosal), and vaccination schedule (homologous and heterologous prime-boost strategies, intervals between doses, etc.) are of critical importance for shaping the host immune response and eliciting the optimal response for a specific pathogen (Harandi and Medaglini, 2010; Ciabattini et al., 2013, 2015, 2016, 2018b; Fiorino et al., 2013; Cunningham et al., 2016; Dacoba et al., 2017; Handel et al., 2018). Vaccine immune response is also deeply affected by the host immune system, especially at the extremes of life, when early life immune immaturity or age-associated immune alteration is present (Mohr and Siegrist, 2016; Ciabattini et al., 2018a). In the elderly, the efficacy of vaccination is indeed strongly reduced than in younger adults, mostly owing to alteration of their immune system, where some immunological components decline whereas others, such as inflammation, are increased (Ciabattini et al., 2018a). Because the intestinal microbiota plays a crucial rule in the regulation of the immune system, it can be considered as another factor that might affect how individuals respond to vaccinations (Zimmermann and Curtis, 2018b). Microbial community composition is in turn affected by age, environmental and socio-economic factors, diet, gender, chronic infections, immunosuppressive chemotherapy, antibiotic treatment, or probiotic use (Conlon and Bird, 2014; Becattini et al., 2016; Thursby and Juge, 2017; Vemuri et al., 2018; Zimmermann and Curtis, 2018a; Figure 2). Variations in microbial communities, due to environmental, socioeconomic, nutritional, and hygiene conditions, could in part explain the geographical heterogeneity in vaccine responses (Velasquez et al., 2018).

Even though the specific mechanisms by which the microbiota affects vaccine responses are not completely understood, it has been demonstrated that the microbiota constitutes a constant source of natural adjuvants capable of activating a multitude of pathways that control innate and adaptive immunity (Pabst and Hornef, 2014). This “endogenous adjuvant potential” was demonstrated in a study on inactivated influenza vaccine that showed that GF or antibiotic-treated mice had significantly impaired antibody responses to this vaccine (Nakaya et al., 2011; Oh et al., 2014). The study demonstrated a strong correlation between the expression of TLR5 and the magnitude of the antibody response, which was significantly reduced in TLR5-deficient mice immunized with TIV than in wild-type mice. By using GF mice and antibiotics treatment, it was demonstrated that commensal bacteria were the source of TLR5 ligands responsible for the enhancement of immune response to TIV. Oral reconstitution with a flagellated strain of *Escherichia coli* was sufficient to restore the normal antibody response. Similar results were observed also toward the inactivated poliovirus vaccine (Oh et al., 2014), but not toward other vaccines such as adjuvanted vaccine against tetanus–diphtheria–pertussis or a live-attenuated yellow fever vaccine (YF-17D), demonstrating that many factors, such as vaccine formulation components and route of immunization, influence the relation between microbiota and immune response (Oh et al., 2014).

Besides flagellin, other components of the microbiota have shown an adjuvant role in the induction of the immune response to vaccination. The peptidoglycan component muramyl dipeptide (MDP), agonists of nucleotide-binding oligomerization domain containing 2 (Nod2) sensor, has been shown to enhance the adjuvanticity of cholera toxin in the nasal cavity of mice (Kim et al., 2016). The depletion of bacteria by antibiotic treatment suppressed the induction of the humoral response following mucosal immunization, whereas reconstitution of GF mice with a Nod2 agonist promoted robust CT adjuvant activity, thus confirming that the microbiota influences mucosal adjuvant activity (Kim et al., 2016). Similarly, the use of monophosphoryl lipid A (MPL), a component of bacterial LPS recognized by the TLR4, enhanced the adaptive responses during vaccination (Duthie et al., 2011).

It has recently been shown that antibiotic-driven dysbiosis in early life of mice leads to impaired antibody responses to five different adjuvanted and live vaccines frequently administered to infants worldwide including the meningococcal serogroup B vaccine (Bexsero); the meningococcal serogroup C vaccine (NeisVac-C); the 13-valent pneumococcal conjugate vaccine (Prevenar); the hexavalent combination vaccine against hepatitis B, diphtheria, tetanus, pertussis, *Haemophilus influenzae* type b, poliomyelitis virus (INFANRIX Hexa); and the tuberculosis BCG vaccine (Lynn et al., 2018). In all cases, early exposure to antibiotics resulted in impaired antibody responses, while T-cell cytokine production was not reduced. Restoration of the commensal microbiota following antibiotic exposure rescued these impaired responses. In contrast, antibiotic-treated adult mice did not exhibit impaired antibody responses to vaccination. Therefore, the impairment of humoral responses seemed to be dependent on antibiotic-driven dysbiosis rather than on direct effects of the antibiotics themselves.

Clinical trials exploring the association of the gut microbiota with vaccine response in humans or comparing the fecal microbiota composition and diversity of responders to non-responders have been performed in high- and low-income countries across diverse ages. The potential impact of the gut microbiota has been investigated with systemic vaccines (Huda et al., 2014), but mostly with oral vaccines, such as oral RVV, oral polio vaccine (OPV), and cholera in infants/children living in low-income countries (Levine, 2010; Magwira and Taylor, 2018). The comparison of the pre-vaccination fecal microbiome composition of RVV responders and non-responders in Ghanaian and Pakistani infants supported a correlation between microbiome composition and RVV immunogenicity (Harris et al., 2017; Harris V. et al., 2018). Bacteria related to *Streptococcus bovis* species were more abundant in Ghanaian responders than in non-responders, resulting in a significant positive association with RVV efficacy, whereas *Bacteroides* and *Prevotella* species, more represented in the microbiome of non-responders correlated with a lack of RVV response (Harris et al., 2017). Similarly, in Pakistani infants, RVV response positively correlated with increased ratio of Gram-negative over Gram-positive bacteria, notably reflected in an approximately three-fold increased abundance of the Proteobacteria related to *Serratia* and *E. coli* (Harris V. et al., 2018). The intestinal microbiota of matched Dutch infants, with high RVV immune responses, showed also a higher abundance of Proteobacteria, particularly Gammaproteobacteria, which include bacteria related to *Serratia* and *E. coli*. Gram-negative bacteria, such as Proteobacteria, can stimulate innate immune responses, such as through their expression of flagella or toxigenic LPS. Proteobacteria or their cell envelope components may therefore be acting as natural immune adjuvants in the Pakistani infant population. These studies provide relevant insights to deepen the causative association between microbiome composition and RVV immunogenicity, and offer a model for studying how the immunogenic potential of the microbiota could be exploited to improve vaccine immunogenicity (Harris, 2018). The modulation of the resident microbial community through the treatment with antibiotics has also been investigated as possible strategy to increase RVV immunogenicity (Harris V.C. et al., 2018).

Supplementation with probiotics is another strategy assessed for improving vaccine immunogenicity by modulating the resident microbiota. Probiotics are defined as live microorganisms, which are beneficial to the host when administered orally in adequate amounts (thought to be ≥10^9^ colony-forming units daily) (Hill et al., 2014). The most frequently used microorganisms are *Lactobacillus* spp., *Bifidobacterium* spp., and *Saccharomyces boulardii*. Recently, two systematic reviews have extensively analyzed the possible influence of probiotics on oral (Church et al., 2019) or both oral and parenteral (Zimmermann and Curtis, 2018a) vaccine responses. A total of 26 studies investigating the use of 40 different probiotic strains on the efficacy of 17 different vaccines (DTP, DTwP, DTaP-Hib, DTaP-IPV-Hib, HAV, HBV, Hib, LAIV, MMRV, OCV, OPV, ORV, PCV7, PPV23, polio, TIV, and Ty21a) were reviewed (Zimmermann and Curtis, 2018a). The meta-analysis concluded that a beneficial effect of probiotics was reported in about half of the studies, and it was strongest for oral vaccinations and for parenteral influenza vaccination, suggesting a possible beneficial role in elderly people, in whom it is known that seroconversion rates to influenza vaccination are lower than in younger people. No significant benefits on the immune response to oral vaccination were instead reported in the analysis performed by Church, who analyzed a total of four oral studies (Church et al., 2019). Further studies are needed to fully elucidate the role of probiotics on modulating vaccine immune responses, and specific focus should be on establishing optimal strains, doses, and timing of administration.

The impact of environmental enteropathy (Gilmartin and Petri, 2015)—a microbiota alteration associated with chronic intestinal inflammation due to the increased exposure of infants living in resource-poor settings to fecal–oral bacteria through contaminated water and food—has also been investigated in oral vaccination (Kirkpatrick et al., 2015; Parker et al., 2018). No inhibitory effect of enteropathogens on vaccine response was reported, even though the differences in the methodologies used for microbiome analysis with respect to those of other studies can impact the results observed. Also, the small bowel bacterial overgrowth (SBBO) elicited by the presence in the small bowel of fecal bacterial species usually restricted to the large bowel has been investigated as a possible factor that impaired vaccine response to cholera vaccine CVD 103-HgR (Lagos et al., 1999). The hypothesis is that live oral vaccines might be destroyed by an already activated innate system with consequently poor induction of specific immune responses by antigens enclosed in the vaccines (Levine, 2010).

The potential role of the microbiota on the immune response to parenteral vaccination has been investigated in Bangladeshi infants (Huda et al., 2014). Actinobacteria were the most abundant phylum recovered, followed by Firmicutes, Proteobacteria, and Bacteroidetes. Within the phylum of Actinobacteria, the most abundant genera were *Bifidobacterium*, and the subspecies *Bifidobacterium infant* predominated. A positive association between *Bifidobacterium* and some adaptive immunological responses was observed, such as CD4^+^ and CD8^+^ T-cell proliferative responses to PPD and TT, the delayed-type hypersensitivity to PPD, and specific IgG responses to TT and hepatitis B (HBV) vaccines, whereas high levels of enteric pathogens such as Enterobacteriales and Pseudomonadales were associated with neutrophilia and lower vaccine responses (Huda et al., 2014).

A summary of the different pre-clinical and clinical studies performed for dissecting the intersection between intestinal microbiota and vaccine response is reported in Table 1.

An important technical challenge when studying the role of microbiota on the immune response to vaccines is related to the methods used for identifying fecal or intestinal microbial composition. Recently, bioinformatics approaches and high-throughput bacterial genome sequencing have significantly improved our knowledge of the role of the microbiota in health and disease, as described in the next paragraph. In-depth analysis of the impact of the microbiota on vaccine responses in humans, using the latest technologies, is instrumental for identifying strategies to improve vaccine efficacy and duration of protection.

## New Tools to Study the Impact of the Microbiome on the Immune Response to Vaccination

**FIGURE 3:**
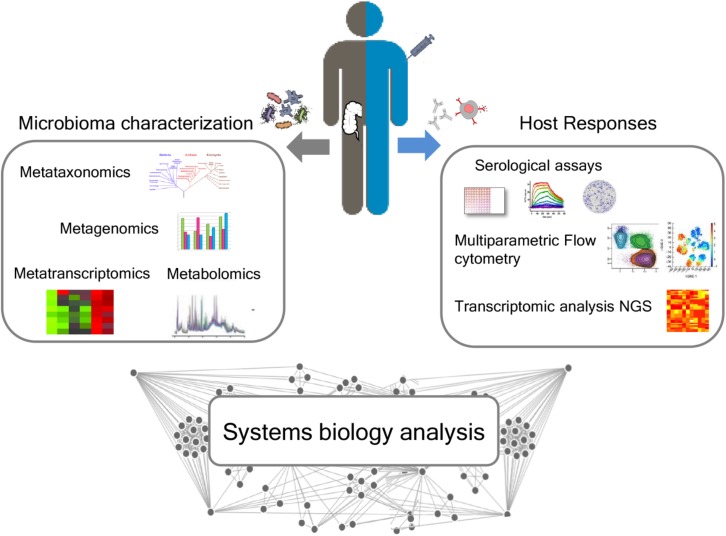
Integrated approach for analyzing the relationship between microbiota and immune response following vaccination. A deep characterization of the gut microbiome can be obtained by NGS approaches, including metataxonomics, metagenomics, metatranscriptomics, and metabolomics. Advanced technologies, including multiparametric flow cytometry and transcriptomic analysis, allow to profile both the humoral and cellular immune responses. A systems biology integration of microbiome characterization with host response analysis upon vaccine administration could allow to better correlate the influence of the intestinal microbiome on vaccine responses.

The influence of gut microbiota on vaccine immune responses can be evaluated by an integrated approach between microbiome characterization and vaccine immune response through a systems biology approach. The availability of novel tools to deeply analyze the microbiome, such as next-generation sequencing (NGS) technologies, and the host immune response are paving the way for elucidating the impact of the microbiome in modulating the vaccine immune response (Figure 3).

Recently, NGS technologies such as Illumina, Ion Torrent (Life Technologies), Pacific Biosciences, Nanopore Sequencing (Oxford Nanopore Technologies), and the bioinformatics tools developed to interpret sequence data have revolutionized the characterization of microbial communities, overcoming some limitations of the culture-based methods leading to an improved and deeper understanding of the microbial population within an ecosystem. Sequence-based methods provide information for identifying microorganisms and outlining their physiological functionality, whereas classical culturing techniques are limited to the study of phenotypic characteristics.

NGS approaches include 16S rRNA gene sequencing, metagenomics, and metatranscriptomics. The majority of microbiome studies characterize bacteria present in a sample by sequencing a fragment of the 16S rRNA encoding gene, a process called metataxonomics (Marchesi and Ravel, 2015). The 16S rRNA gene has the advantage of containing highly conserved regions whose sequence is conserved in most bacterial species and nine hypervariable regions (V1–V9) whose sequence is specific for a given species. Deep sequencing of these hypervariable regions is used to define the composition of bacterial communities and to compare communities over time or across variable conditions such as antibiotic treatments (Faust and Raes, 2012). Long-read sequencing of the 16S rRNA gene is a promising approach to provide high-resolution analysis of microbial communities at the species level, too. Metataxonomics represents a powerful tool for the analysis of the microbiota, improving taxonomy resolution to the strain level.

Whereas the marker gene-specific approach allows for the investigation of microbial diversity in an environment by a common marker gene set, whole-genome sequencing (WGS) provides sequencing of genomic DNA extracted from the entire microbial community to catalog all the genetic materials present in a sample. This approach allows for a global picture of microbial population, producing an unbiased representation of the complete set of microbial genes (Sharpton, 2014; Aguiar-Pulido et al., 2016).

DNA-sequencing-based approaches cannot generally distinguish between living and metabolically active, damaged, or dead bacterial cells, or free DNA, providing a partial vision of the functional profile of a microbial community. RNA sequencing analyses can identify living and metabolically active cells, informing on transcriptional activity of the community. Knowledge of the transcriptome of a given organism offers significant insights into cellular processes and allows the identification of proteins that may not be produced or expressed under current culture assay conditions.

The development of new “omics”-based technologies has made possible to rapidly investigate the diversity of microbial populations and at the same time to characterize the relative abundance of all taxa and functions in a defined community (Jansson et al., 2012). As for metagenomics, it is now possible to perform whole transcriptome shotgun sequencing. Metatranscriptomics, in fact, involves the complete sequencing of the transcriptome (coding and non-coding RNAs) and provides a deeper understanding of the functional output of the genome and valuable insight into gene expression patterns, gene function, and regulation (van Vliet, 2010; Gilbert and Hughes, 2011). Combined approaches that provide information on both identity and physiology of the bacteria in a community can improve the characterization of the microbiome.

In this view, metabolomics provides identification and quantification of all metabolites (small molecules released by the organism into the immediate environment) produced by a microorganism or collectively by a microbial community of a sample. The metabolome (the set of all metabolites present in any given strain or microbial community) represents an important evidence of community function and is considered the most direct indicator of the homeostasis alteration in a specific environment (such as dysbiosis). Metabolomics studies the consequences of the shifts in the collective gene expression of the microbial community that modifies the medium where the microbial community must feed, grow, reproduce, and cooperate or compete to survive (Aguiar-Pulido et al., 2016). The most common platforms used to characterize the metabolome are nuclear magnetic resonance (NMR) spectroscopy and mass spectrometry (MS) linked to a liquid chromatography separation system (Jansson et al., 2012). The application of these approaches can revolutionize the study of complex interactions between the microbiota and the immune system.

At the same time, the advancement of multiple technological platforms to examine the host immune response has allowed to profile the humoral and cellular immune responses to vaccination. Promising approaches used to measure immunity induced following vaccination include antibody-profiling technologies, high-throughput flow cytometry, transcriptomic analysis (Furman and Davis, 2015). Novel analytical approaches, able to integrate diverse facets of the humoral immune response and define the relationships between antibody populations and functions, are now available. These approaches reveal features of vaccine-induced “fingerprints” and offer new insights concerning polyclonal antibody immune responses elicited by vaccines (Chung et al., 2015; Kawahara et al., 2019).

Measure of the frequency and functions of antigen-specific lymphocytes can be obtained by multiparametric flow cytometry employing antibody panels covering the simultaneous analysis of cell surface markers, multiple cytokines, and other functional markers. High-throughput flow cytometry allows to dissect both B and T immune responses. For example, antigen-specific CD4^+^ T lymphocytes, which play a fundamental role in the adaptive immune response upon vaccination, can be detected and characterized by MHC class II–peptide complex tetramers (Crawford et al., 1998; Prota et al., 2015) and their effector function, that is, cytokine production, can be assessed (Huang et al., 2013; Pastore et al., unpublished). Novel computational techniques have been developed in the recent years for analyzing, visualizing, and interpreting large amounts of data obtained by multiparametric flow cytometry (Saeys et al., 2016) and can be employed, for example, for better visualizing polyfunctional T cells elicited by vaccination (Ciabattini et al., 2018b).

Nucleic acid sequencing enables a very broad approach to immunoglobulin or TCR repertoire analysis, allowing the determination of the diversity and clonal expansion of lymphocytes responding to vaccine (Galson et al., 2014; VanDuijn et al., 2017). The NGS methodology has become a core technology in vaccine analysis (Dhiman et al., 2009; Santoro et al., 2018) and, integrated with immunological results, has been used to profile the human response to different vaccines. This integrating approach is called “systems biology” and can be used to analyze multiple data types related to complex biological interactions by using computational analysis and mathematical modeling and to predict vaccine protection and immunogenicity (Querec et al., 2009; Li et al., 2014; Nakaya et al., 2016; Kazmin et al., 2017; van den Berg et al., 2017).

A systems biology integration of microbiome characterization with host response analysis upon vaccine administration could allow to better correlate the influence of the intestinal microbiome with vaccination (Figure 3).

## Challenges in the Study of the Potential Role of the Microbiota in Vaccine Immune Response

The characterization of the potential role of the microbiome on vaccine immunogenicity might help in the tailoring of vaccination strategies; nevertheless, there are some challenging factors that need to be properly addressed.

The application of culture-independent techniques, such as metagenomics and metatranscriptomics, have expanded the experimental tools available for studying the microbiome, providing information on the taxonomic profile of a microbial community and contributing to the characterization of the dynamics of functional profiles with varying conditions (Aguiar-Pulido et al., 2016). Furthermore, transcriptomic and metabolomic analysis of the host responses can provide important insights on the impact of microbial community on vaccine response. The application of systems biology approaches offers important opportunities to integrate data related to both microbiome and the host vaccine response. A critical challenge remains in the identification of microbiome targets correlating with vaccine immunogenicity.

Another critical aspect is the availability of reliable animal models with defined microbial composition to explore host–microbe interactions. Gnotobiotic models, including GF, or antibiotic-treated mice have provided substantial insights on the interplay of the microbiota and host immune system (Smith et al., 2007), identifying key microbes responsible for the development of the intestinal immune system (Umesaki and Setoyama, 2000), and have the potential to tell us much about the impact of the microbiota on the vaccine responses (Fiebiger et al., 2016; Cram et al., 2018). However, the absence of microbiota in GF mice or the reduced microbial richness and diversity in mouse models with a defined microbiota does not fully represent the complex interactions that occur within the microbiota of conventionally raised mice; therefore, results from gnotobiotic mice should be interpreted with caution. Moreover, the murine microbiota is influenced by various factors such as breeding environment, interchange variability, genetic backgrounds, diet, sex, and age (Nguyen et al., 2015; Miyoshi et al., 2018). To minimize these effects and improve consistency and reproducibility in murine microbiota studies, procedures such as using a single genetically identical strain, housing all animals in one specific room (to control environment), using the same diet, and utilizing age- and sex-matched mice should be employed (Miyoshi et al., 2018).

In human studies, the large inter-individual variability of the microbiota together with the genetic diversity is a confounding factor that is challenging to overcome when studying host–microbe interactions. Several studies that aimed to assess the impact of the microbiome in vaccine response have been conducted in geographically different populations with different microbiome compositions, as performed in low- and high-income countries (Harris et al., 2017; Harris V. et al., 2018). Another approach is based on altering the microbiota composition with antibiotic treatment to promote amplification or reduction of specific bacterial species prior to vaccine administration (Harris V.C. et al., 2018). This model could be highly informative even if limitations such as the off-target effects of antibiotics and their possible impact on immune responses as well as the impact of the antibiotic spectrum could influence the study readouts (Harris V.C. et al., 2018).

Furthermore, the majority of vaccinations are given during the first few years of life when human immune response and the microbial community develop in concert and are reciprocally influenced. Therefore, careful attention should be given to understanding how the early microbiome affects vaccination and how vaccination may influence the development of the microbiota itself (Jamieson, 2015). Moreover, since there are significant differences in the intestinal microbiota in infants or children compared with adults (Yatsunenko et al., 2012), studies of microbiota and vaccine immune response should be performed in different age groups, even though infant studies are more challenging.

An additional bottleneck is that currently most of the clinical studies performed can only assign correlations but not causality, and the correlations have been made with immunogenicity and not vaccine efficacy (Harris, 2018).

## Conclusions

Vaccine efficacy is based on the capacity of the antigen to elicit a protective immune response and on the immune system competence to respond appropriately to an antigenic stimulation. Recent studies suggest that the microbiota could represent a key element potentially capable to affect both these functions by acting as immunologic modulator as well as natural vaccine adjuvant. The mechanisms underlying the crosstalk between the gut microbiota and immune system are of critical importance especially in early life when the majority of vaccinations are given. During the first years of life, the early gut microbiota can shape immunologic functions and vice versa owing to both the plasticity of the immune system and at the same time the instability of the microbiota. This interaction, together with other genetic and environmental factors, results in a defined microbiota composition and richness that can diversify the individual response to vaccinations. Variations in microbial communities may in part explain the geographical heterogeneity in vaccination success, and a deep understanding of these dynamics could provide a tool to improve immunization strategies. There are evidences of the low response to oral vaccines in low-income countries, and it has been supposed that differences in the intestinal microbiota composition due to environmental, socioeconomic, nutritional, and hygiene conditions could justify these discrepancies. These findings make it reasonable to speculate a hypothetical role of the microbiota in vaccine non-responders. To date, few studies analyze the mechanism by which the microbiota influences the immunogenicity of vaccines, and the results do not show a direct causal link between specific community composition and responsiveness. But interestingly, some bacterial species or their specific components emerge as potent modulators of humoral or cellular immunity among vaccine responder subjects. In this context, different approaches are used to test the potential adjuvant effect of certain probiotic strains to enhance immune response to both oral and parenteral vaccines with discordant results. Although these studies suggest an important role of the microbiota in the modulation of vaccine immune responses, there is the need for further investigations to clearly demonstrate this intersection. The availability of advanced technologies for characterizing both the microbial community and the immunological responses integrated trough a systems biology approach offers the opportunity to deeply analyze the interplay of the microbiota with the immune response to vaccination.

## Author Contributions

AC and RO wrote the article and performed all the necessary literature searches and data compilation. EL contributed to the sections on “Human Gut Microbiota” and “New Tools for to Study Microbiome Impact on Vaccine Immune Response.” DM conceived and critically revised the review article. All authors have read and approved the final manuscript.

## Conflict of Interest Statement

The authors declare that the research was conducted in the absence of any commercial or financial relationships that could be construed as a potential conflict of interest.
